# A multi-database pharmacovigilance study reveals distinctive immunosuppressive and opportunistic infection disproportionality signals with bevacizumab and temozolomide combination therapy in glioblastoma

**DOI:** 10.3389/fmed.2026.1773599

**Published:** 2026-03-11

**Authors:** Yong Yu, Xiaohong Hou, Kaya Xu

**Affiliations:** 1 Department of Neurosurgery, The Second People’s Hospital of Guiyang (Jinyang Hospital), The Affiliated Jinyang Hospital of Guizhou Medical University, Guiyang, Guizhou, China; 2 Department of Neurosurgery, Affiliated Hospital of Guizhou Medical University, Guiyang, Guizhou, China

**Keywords:** bevacizumab, glioblastoma, multi-database, pharmacovigilance, temozolomide

## Abstract

**Background:**

Bevacizumab (BEV) plus temozolomide (TMZ) is increasingly used for glioblastoma, yet the immunosuppression-related adverse event spectrum and safety signals of this combination—including potential interaction effects and time-to-onset patterns—have not been comprehensively characterized in real-world pharmacovigilance data.

**Methods:**

A retrospective analysis was conducted using FAERS and CVARD; a multi-algorithm framework (Omega shrinkage model, PRR, and ROR) was applied for signal detection, and interaction analyses were conducted to explore potential drug–drug interaction signals (more-than-additive reporting disproportionality patterns) in spontaneous reporting data; the Weibull model was used to evaluate time-dependent onset-hazard patterns based on TTO, and multivariate logistic regression was performed to identify factors associated with IRAE reporting.

**Results:**

A total of 1,076 reports in the BEV + TMZ combination therapy group, 2,633 reports in the BEV monotherapy group, and 3,156 reports in the TMZ monotherapy group were included. The combination regimen exhibited a unique IRAEs profile, showing strong reporting disproportionality signals for rare but serious immunosuppressive and opportunistic infection events, including hemophagocytic lymphohistiocytosis (HLH; *Ω* = 0.648), strongyloidiasis (*Ω* = 0.744), Epstein–Barr virus infection (*Ω* = 0.580), and cytomegalovirus pneumonitis (*Ω* = 0.943). Drug interaction analysis suggested potential interaction signals between BEV and TMZ in spontaneous reporting data, with markedly elevated PRR/interaction ORs for hematological (e.g., pancytopenia) and non-hematological toxicities (e.g., enteritis), exceeding the additive effect expected from the two monotherapies in spontaneous reporting data. Temporal dynamic analysis indicated that combination therapy altered the time-to-onset pattern for some events; for instance, the median time to onset for lymphocytopenia shifted from 34 days (BEV monotherapy) to 23 days, and the onset-hazard pattern shifted from an increasing type to a decreasing type. Multivariable logistic regression adjusting for sex, age group, body-weight group, and reporter type showed higher odds of IRAE reporting for BEV monotherapy versus TMZ monotherapy (OR = 3.6037, 95% CI: 1.0378–12.652, *p* < 0.05), while the overall IRAE reporting odds for BEV + TMZ were not significantly different from TMZ (OR = 2.9677, 95% CI: 0.5018–15.2056, *p* = 0.1988) or BEV (OR = 0.8235, 95% CI: 0.1454–4.6642, *p* = 0.8263).

**Conclusion:**

BEV + TMZ combination therapy shows disproportionality signals for severe immunosuppression and opportunistic infections. These signal patterns, including time-to-onset distributions, may help prioritize clinical vigilance and hypothesis generation, but do not estimate incidence or establish causality and require prospective confirmation.

## 1 Introduction

Glioblastoma (GBM) is one of the most malignant primary brain tumors with the poorest prognosis, and its treatment remains a significant challenge (1). Bevacizumab (BEV), a monoclonal antibody targeting vascular endothelial growth factor (VEGF) (2), and temozolomide (TMZ), an oral alkylating chemotherapeutic agent, have become important tools in neuro-oncology, particularly in the treatment of recurrent or high-grade gliomas (3, 4). BEV exerts its antitumor effects by inhibiting tumor angiogenesis (5), whereas TMZ induces tumor cell apoptosis via DNA methylation (6). In recent years, the combined treatment regimen of BEV and TMZ has been increasingly utilized in clinical practice, aiming to enhance therapeutic efficacy through potential therapeutic complementarity.

However, with the expanded use of this combination regimen, concerns regarding its safety profile, particularly its potential impact on the immune system, have garnered increasing attention (7, 8). BEV monotherapy is known to be associated with adverse events such as hypertension, proteinuria, hemorrhage, and impaired wound healing (9), while the toxicity profile of TMZ is most notably characterized by myelosuppression (e.g., neutropenia, thrombocytopenia) (10). Although the safety profiles of both individual agents have been well-described, the safety characteristics of their combination are not simply an additive sum of their individual toxicities. The VEGF pathway plays a crucial role in modulating the immune microenvironment (11). Consequently, the combination of the VEGF inhibitor BEV with TMZ, which possesses immunomodulatory potential, may yield a unique spectrum of immune-related toxicities, the specific characteristics and patterns of which remain unclear.

Currently, systematic studies investigating immune-related adverse events (IRAEs) associated with the combination of BEV and TMZ are still relatively scarce. To address this knowledge gap, this study aims to conduct a comprehensive pharmacovigilance analysis utilizing the US FDA Adverse Event Reporting System (FAERS) database and the CVARD database (12, 13). It is anticipated that this research will provide detailed evidence-based support for the safe clinical application of BEV and TMZ, especially in combination, and offer scientific guidance for identifying high-risk patients and developing timely monitoring strategies.

## 2 Data and methods

### 2.1 Data source and preprocessing

**Figure 1:**
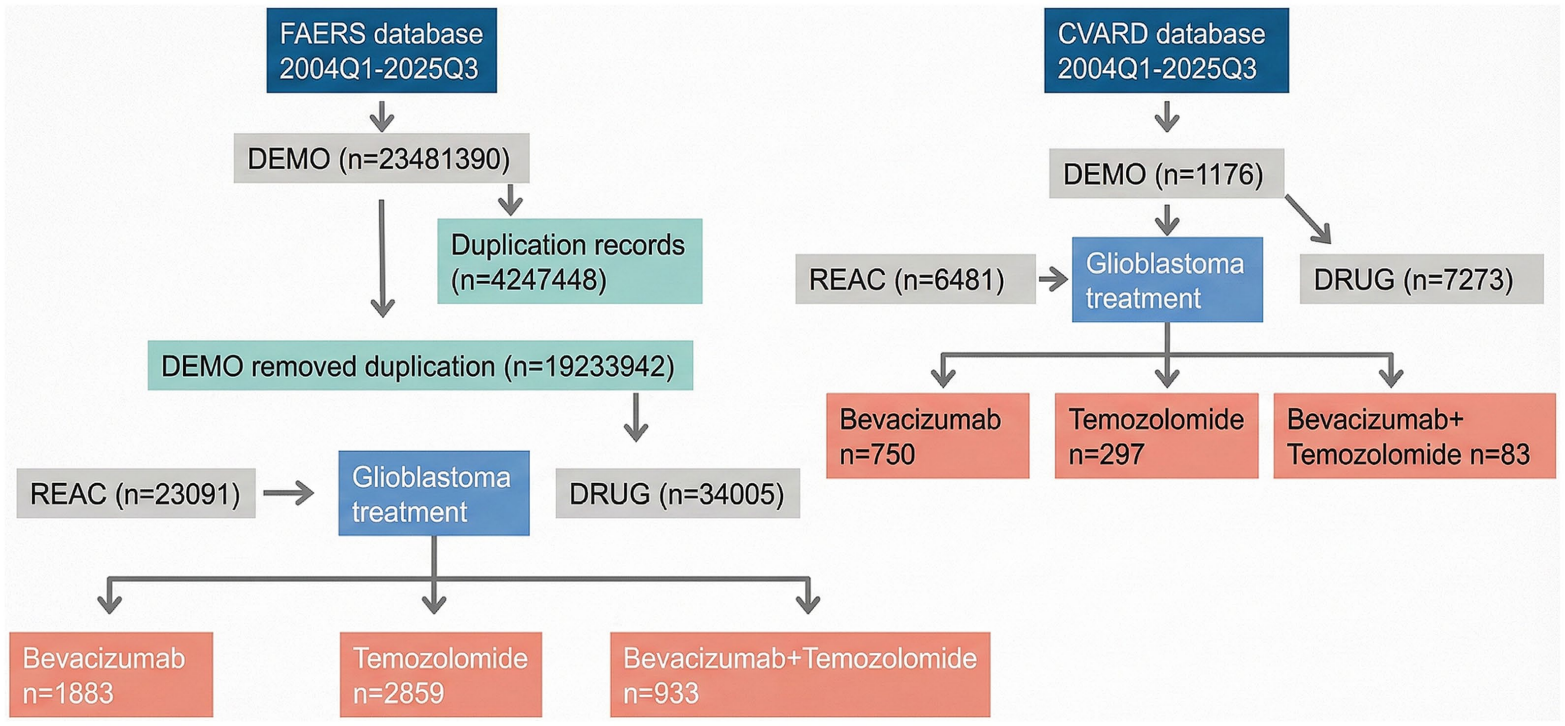
A flowchart for screening immune-related adverse events of bevacizumab and temozolomide from the FAERS and CVARD databases.

This study conducted a retrospective analysis based on the FAERS database (data extraction period: Q1 2004 to Q3 2025; downloaded on [https://www.fda.gov/drugs/fdas-adverse-event-reporting-system-faers/fda-adverse-event-reporting-system-faers-public-dashboard]) and the CVARD database (data extraction period: January 2004 to October 2025; downloaded on [https://cvp-pcv.hc-sc.gc.ca/arq-rei/?lang=eng]). The data preprocessing procedure included: (1) extracting variables such as demographic information, adverse event reports, suspect drugs, and other concomitant medications from the raw data of each database; (2) identifying and removing duplicate reports according to the FDA-recommended procedure; (3) standardizing adverse event terms using the Medical Dictionary for Regulatory Activities (MedDRA, version [28.0]). Drug exposure in the primary analysis was defined using the reporter-designated “Primary Suspect” (PS) role. Monotherapy reports were defined as those in which BEV (or TMZ) was recorded as PS and the other study drug was not listed in the same report. Combination therapy reports were defined as those listing both BEV and TMZ, with at least one recorded as PS and the other recorded as a concomitant medication (C) (i.e., BEV=PS and TMZ = C, or TMZ = PS and BEV = C). To assess robustness to potential misclassification from reporter-assigned drug roles, we conducted a sensitivity analysis using an expanded “any-role” exposure definition that included PS, Secondary Suspect (SS), Concomitant (C), and Interacting (I) roles (PS/SS/C/I) and re-evaluated key prioritized signals (Supplementary Table S2). To restrict analyses to glioblastoma, indication/diagnosis text fields were normalized and queried using a pre-specified dictionary of GBM-related terms. The primary cohort used broad GBM-related terms, and a more stringent “core GBM” subgroup was defined *a priori* (Supplementary Table S1). Reports with missing or non-matching indication/diagnosis fields were excluded (Figure 1).

### 2.2 Patient baseline characteristics and reporting overview

Descriptive statistical analysis was performed on the baseline characteristics of all included patient reports. Categorical variables (e.g., sex, age group, weight group, reporter type) are presented as frequency (*N*) and percentage (%), used to describe the distribution of demographic characteristics and reporting patterns across the different treatment groups. Inter-group differences in baseline characteristics (including missing/unknown categories) were formally compared using Pearson’s chi-square tests, and the corresponding *p* values are reported in Tables 1, 2.

**Table 1:** Basic characteristics of reports grouped according to reported drug use combinations in the FAERS database.

BEV + TMZ	*N* (%)	BEV	*N* (%)	TMZ	*N* (%)	*p* value
Sex		Sex		Sex		4.75 × 10^−22^
Male Female Unknown	400 (42.9%) 340 (36.4%) 193 (20.7%)	Male Female Unknown	978 (51.9%) 603 (32.0%) 302 (16.0%)	Male Female Unknown	1,358 (47.5%) 1,206 (42.2%) 295 (10.3%)	
Weight		Weight		Weight		
<50 kg 50–100 kg >100 kg Unknown	15 (1.6%) 237 (25.4%) 22 (2.4%) 659 (70.6%)	<50 kg 50–100 kg >100 kg Unknown	21 (1.1%) 630 (33.5%) 94 (5.0%) 1,138 (60.4%)	<50 kg 50–100 kg >100 kg Unknown	38 (1.3%) 681 (23.8%) 51 (1.8%) 2089 (73.1%)	2.59 × 10^−21^
Age		Age		Age		
<2 years 2–11 years 12–17 years 18–64 years 65–85 years >85 years Unknown	1 (0.1%) 7 (0.8%) 7 (0.8%) 468 (50.2%) 220 (23.6%) 1 (0.1%) 229 (24.5%)	<2 years 2–11 years 12–17 years 18–64 years 65–85 years >85 years Unknown	1 (0.1%) 9 (0.5%) 12 (0.6%) 948 (50.3%) 268 (14.2%) 1 (0.1%) 644 (34.2%)	2–11 years 12–17 years 18–64 years 65–85 years >85 years Unknown	21 (0.7%) 28 (1.0%) 1,464 (51.2%) 800 (28.0%) 4 (0.1%) 542 (19.0%)	9.64 × 10^−38^
Reported person		Reported person		Reported person		
CN HP MD OT PH Unknown	130 (13.9%) 220 (23.6%) 411 (44.1%) 143 (15.3%) 11 (1.2%) 18 (1.9%)	CN HP MD OT PH LW Unknown	335 (17.8%) 310 (16.5%) 789 (41.9%) 319 (16.9%) 98 (5.2%) 1 (0.1%) 31 (1.6%)	CN HP MD OT PH RN Unknown	218 (7.6%) 652 (22.8%) 1,165 (40.7%) 573 (20.0%) 155 (5.4%) 4 (0.1%) 92 (3.2%)	1.90 × 10^−32^

*p* values were calculated using Pearson’s chi-square tests across the three regimens (BEV+TMZ, BEV, TMZ) within each database; missing/unknown values were retained as explicit categories.

**Table 2:** Basic characteristics of the population grouped according to reported drug use combinations of CVARD database.

BEV + TMZ	*N* (%)	BEV	*N* (%)	TMZ	*N* (%)	*p* value
Sex		Sex		Sex		
Male Female Unknown	38 (45.8%) 39 (46.4%) 7 (8.4%)	Male Female Unknown	446 (59.5%) 295 (39.3%) 9 (1.2%)	Male Female Unknown	176 (59.3%) 107 (36.0%) 14 (4.7%)	3.98 × 10^−5^
Weight		Weight		Weight		
50–100 kg >100 kg Unknown	42 (50.6%) 5 (6.0%) 36 (43.4%)	<50 kg 50–100 kg >100 kg Unknown	12 (1.6%) 334 (45.9%) 63 (8.4%) 331 (44.1%)	<50 kg 50–100 kg >100 kg Unknown	2 (0.7%) 25 (8.4%) 2 (0.7%) 268 (90.2%)	4.15 × 10^−38^
Age		Age		Age		
18–64 years 65–85 years Unknown	63 (75.0%) 8 (9.6%) 13 (15.7%)	12–17 years 18–64 years 65–85 years Unknown	2 (0.3%) 591 (78.8%) 82 (10.9%) 75 (10.0%)	12–17 years 18–64 years 65–85 years Unknown	3 (1.0%) 176 (59.3%) 22 (7.4%) 96 (32.3%)	1.35 × 10^−15^
Reported person		Reported person		Reported person		
Consumer Health professional Physician Unknown	13 (15.7%) 23 (27.7%) 8 (9.6%) 40 (47.6%)	Consumer Health professional Physician Pharmacist Unknown	67 (8.9%) 182 (24.3%) 78 (10.4%) 8 (1.1%) 415 (55.3%)	Consumer Health professional Physician Pharmacist Unknown	175 (58.9%) 34 (11.4%) 61 (20.5%) 7 (2.4%) 20 (6.7%)	5.26 × 10^−81^

*p* values were calculated using Pearson’s chi-square tests across the three regimens (BEV+TMZ, BEV, TMZ) within each database; missing/unknown values were retained as explicit categories.

### 2.3 Signal detection and drug interaction analysis

A multi-level statistical framework was employed for the detection and validation of adverse event signals. The *Ω* shrinkage model was primarily utilized for signal mining, as this Bayesian method effectively handles sparse data and provides more robust disproportionality estimates. Concurrently, the Proportional Reporting Ratio (PRR) and the Reporting Odds Ratio (ROR) were calculated for methodological triangulation. The signal detection criteria were as follows: for the *Ω* shrinkage model, a signal was identified if the lower limit of the 95% confidence interval (CI) for the *Ω* value was >0 and the number of cases was ≥3; for PRR, a signal was defined as PRR ≥ 2, *χ*^2^ ≥ 4, and the number of cases ≥3; for ROR, a signal was identified if the lower limit of the 95% CI for the ROR was >1 and the number of cases was ≥3. Signal consistency was assessed as follows: strong consistency was defined when all three methods met the significance thresholds; moderate consistency was defined when the *Ω* model plus either traditional method (PRR or ROR) met the significance thresholds; weak consistency was defined when only PRR or ROR met the significance thresholds. Signals with moderate or strong consistency were defined as positive signals. Clinical prioritization was graded as: high priority for signals with strong consistency and PRR > 5; medium priority for signals with strong consistency and PRR > 2, or moderate consistency and PRR > 5; low priority for signals with moderate consistency and PRR ≤ 5, or weak consistency (14, 15). Drug interaction analysis was performed using a multi-algorithm approach to evaluate potential interaction signals in combination therapy within spontaneous reporting data. Because interaction metrics in SRS reflect reporting disproportionality rather than biological interaction, we used the following pre-specified, heuristic prioritization criteria to rank signals for clinical attention. A high-priority interaction signal was defined when all of the following were met: PRR(combination vs. BEV) ≥ 2.0, PRR(combination vs. TMZ) ≥ 2.0, interaction OR ≥ 3.0, and PRR values for both monotherapy groups <1.5 (with 95% CI not including 1), recognizing that very large estimates may occur under sparse cell counts. A moderate-priority interaction signal was defined by meeting any of the following: PRR(combination vs. BEV) ≥ 1.8 and PRR(combination vs. TMZ) ≥ 1.8; or an interaction OR between 2.0 and 2.9, with the 95% CI for at least one comparison not including 1. Drug interaction analysis was performed to explore potential drug–drug interaction signals (more-than-additive reporting disproportionality patterns) in spontaneous reporting data. Because interaction metrics in spontaneous reporting systems reflect reporting patterns rather than biological interaction and can become unstable under sparse cell counts, PRR (combination vs. each monotherapy) and the interaction OR were used as descriptive, hypothesis-generating measures for signal prioritization. We therefore emphasize the presence/consistency of patterns across comparisons and databases, and we avoid interpreting the magnitude of extreme interaction OR estimates as effect sizes or evidence of pharmacodynamic synergy. We prioritized signals when PRR(combination vs. BEV) and PRR(combination vs. TMZ) were both elevated (e.g., ≥2) and the corresponding monotherapy PRRs were not elevated, while treating interaction OR thresholds as supportive rather than definitive.

### 2.4 Time-to-onset analysis and exploratory Weibull shape modeling

Time-to-onset (TTO) was assessed only among reports with non-missing and valid medication start dates and adverse-event dates. TTO was calculated as the number of days between the reported treatment start date and the reported event onset date; reports with missing or implausible dates (e.g., negative intervals) were excluded. Because spontaneous reporting systems do not capture individual follow-up time for exposed patients without the event, right-censoring cannot be defined and Kaplan–Meier/Cox methods are not directly applicable. Therefore, TTO summaries (median, IQR, Min, Max) describe report-based, case-only onset-time distributions among reports with available dates and should be interpreted descriptively. Where report counts were adequate (pre-specified *N* ≥ 10), we additionally applied an exploratory Weibull fit to characterize the shape of the reported onset-time distribution; the Weibull shape parameter (*β*) was used as a descriptive indicator (*β* > 1.2, later-clustering; 0.8–1.2, approximately constant; *β* < 0.8, early-clustering).

### 2.5 Logistic regression analysis for factors associated with IRAE reporting

To evaluate factors associated with immune-related adverse event (IRAE) reporting odds in FAERS, a multivariable logistic regression model was constructed with treatment regimen as the primary exposure. Covariates mandatorily included sex (male as the reference), age group (<65 years as the reference), weight group (50–100 kg as the reference), and reporter type (MD as the reference), with TMZ monotherapy specified as the reference regimen. Results were expressed as odds ratios (ORs) with their 95% confidence intervals (CIs). Model goodness-of-fit was assessed using McFadden’s *R*^2^, Akaike Information Criterion (AIC), and Bayesian Information Criterion (BIC).

### 2.6 Statistical analysis

All data analyses were performed using R software (version 4.4.3). The R scripts used for data cleaning and analysis, together with software environment details (R version and key package versions), are available from the corresponding author upon reasonable request. The statistical significance level was set at a two-sided *p*-value < 0.05 for descriptive and regression analyses only. Because disproportionality screening evaluates a large number of MedDRA preferred terms, we treated all signal-detection outputs as exploratory and subject to signal multiplicity rather than as confirmatory hypothesis tests. To mitigate spurious findings under multiplicity and sparse reporting, we used the *Ω* shrinkage model as the primary estimator, enforced minimum report-count thresholds (*N* ≥ 3), and prioritized signals showing multi-algorithm consistency (*Ω*/PRR/ROR) and, when available, cross-database replication (FAERS and CVARD). All disproportionality metrics (*Ω*/PRR/ROR) quantify reporting associations in spontaneous reporting systems and do not provide incidence estimates due to the absence of exposure denominators. Interaction metrics similarly reflect more-than-additive reporting disproportionality and should be interpreted as potential interaction signals rather than confirmed synergistic toxicity. The logistic regression model evaluates factors associated with IRAE reporting odds, not causal risk.

## 3 Results

### 3.1 Patient baseline characteristics and reporting overview

This study summarized baseline characteristics of spontaneous reports across therapeutic regimens (BEV + TMZ combination therapy, BEV monotherapy, and TMZ monotherapy) from the FAERS and CVARD databases, including sex, body weight, age distribution, and reporter type.

In the FAERS database, the BEV + TMZ combination therapy group comprised 933 cases, the BEV monotherapy group 1,883 cases, and the TMZ monotherapy group 2,859 cases. Regarding gender distribution, in the combination therapy group, males accounted for 42.9%, females for 36.4%, and gender was unknown for 20.7%. A higher proportion of males was observed in the BEV monotherapy group (51.9%), whereas the TMZ monotherapy group had the highest proportion of females (42.2%). The proportion of missing body weight data was generally high, reaching 70.6% in the combination therapy group. Age distribution analysis revealed that patients aged 65–85 years constituted the highest proportion (50.2%) in the combination therapy group, whereas the TMZ monotherapy group was predominantly composed of patients aged 18–64 years (51.2%). Physicians were the primary reporters, accounting for 44.1% of reports in the combination therapy group (Table 1).

In the CVARD database, the BEV + TMZ combination therapy group included 83 cases, the BEV monotherapy group 750 cases, and the TMZ monotherapy group 297 cases. Gender distribution was similar to that in FAERS, with the highest proportion of males observed in the BEV monotherapy group (59.5%). Regarding body weight, 50.6% of patients in the combination therapy group had a body weight between 50 and 100 kg. In terms of age, patients aged 18–64 years constituted 75.0% of the combination therapy group, and this age group also predominated in the BEV monotherapy group (78.8%). Significant differences in reporter type were observed among the treatment groups: the TMZ monotherapy group had the highest proportion of consumer reports (58.9%), whereas physician reports accounted for 47.6% in the BEV + TMZ combination group (Table 2).

Across the three regimens, distributions of sex, age group, body-weight group (with substantial missingness, e.g., 70.6% unknown weight in the BEV + TMZ group in FAERS), and reporter type differed significantly in both FAERS and CVARD (Pearson’s chi-square tests; Tables 1, 2).

### 3.2 Signal detection and comparative analysis of immune-related adverse events for bevacizumab, Temozolomide monotherapy, and combination therapy

Bevacizumab monotherapy: In the FAERS database (Table 3), several significant adverse event signals associated with bevacizumab were identified. Among them, lymphocyte count decreased (*N* = 58, *Ω* = 0.205), arthralgia (*N* = 37, *Ω* = 0.260), and neutrophil count decreased (*N* = 35, *Ω* = 0.245) were characterized not only by high reporting frequencies but also by positive results across multiple signal detection metrics (PRR, ROR). These signals were assessed as having “strong” consistency, suggesting a strong association with the drug, and were thus classified as medium priority for attention. Notably, although diarrhoea (*N* = 60, *Ω* = 0.174) and pruritus (*N* = 27, *Ω* = 0.270) had considerable reporting frequencies, their signal strengths were relatively weak, resulting in a low priority rating. The most prominent signals were optic neuritis (*N* = 6, *Ω* = 0.744) and cholangitis (*N* = 5, *Ω* = 0.921). Despite low absolute report counts, both exhibited extremely high *Ω* values and strong consistency, warranting classification as high-priority signals and suggesting the potential for rare but strongly associated serious adverse events. In contrast, within the CVARD database (Table 4), only one immune-related adverse event with a significant signal was identified: lymphocyte count decreased (*N* = 52, *Ω* = 0.449). This signal was positive across all detection metrics with strong consistency and was classified as medium priority. This finding corroborates the result from the FAERS database, further supporting lymphocytopenia as a noteworthy potential risk associated with bevacizumab. In summary, signal mining results from different databases collectively indicate that ‘lymphocyte count decreased’ is one of the most robust signals associated with bevacizumab. Concurrently, the FAERS database revealed other potential risks, particularly serious events such as ‘optic neuritis’ and ‘cholangitis’ that require heightened vigilance.

**Table 3:** Analysis of immune-related adverse event signals of bevacizumab in the FAERS database.

Adverse event	*N*	*Ω*	95% CI	PRR	ROR	*Ω* Sig	Consistency	Priority
Diarrhoea	60	0.174	(0.06–0.75)	No	Yes	Yes	Moderate	Low
Lymphocyte count decreased	58	0.205	(0.57–1.37)	Yes	Yes	Yes	Strong	Medium
Arthralgia	37	0.260	(0.52–1.54)	Yes	Yes	Yes	Strong	Medium
Neutrophil count decreased	35	0.245	(0.24–1.2)	Yes	Yes	Yes	Strong	Medium
Pruritus	27	0.270	(0.07–1.13)	No	Yes	Yes	Moderate	Low
Optic neuritis	6	0.744	(0.09–3.01)	Yes	Yes	Yes	Strong	High
Cholangitis	5	0.921	(0.09–3.7)	Yes	Yes	Yes	Strong	High

**Table 4:** Analysis of immune-related adverse event signals of bevacizumab in the CVARD database.

Adverse event	*N*	*Ω*	95% CI	PRR	ROR	*Ω* Sig	Consistency	Priority
Lymphocyte count decreased	52	0.449	(0.14–1.9)	Yes	Yes	Yes	Strong	Medium

Temozolomide monotherapy: Temozolomide (TMZ) demonstrated a broader and more intense spectrum of signals in the FAERS database (Table 5), particularly concentrated in the hematological system. Thrombocytopenia (*N* = 402, *Ω* = 0.088), neutropenia (*N* = 177, *Ω* = 0.127), and lymphopenia (*N* = 77, *Ω* = 0.196), characterized by high reporting frequencies, constituted medium-priority signals. In contrast, pancytopenia (*N* = 149, *Ω* = 0.189), febrile neutropenia (*N* = 66, *Ω* = 0.240), and aplastic anaemia (*N* = 24, *Ω* = 0.841) were classified as high priority due to their severe clinical consequences and high signal strength. Furthermore, TMZ was associated with a series of rare but extremely strong signals for serious events, all marked as high priority, including: toxic epidermal necrolysis (*N* = 16, *Ω* = 0.589), drug reaction with eosinophilia and systemic symptoms (*N* = 10, *Ω* = 0.873), progressive multifocal leukoencephalopathy (*N* = 10, *Ω* = 0.873), and pleural effusion (*N* = 13, *Ω* = 0.600). The results from the CVARD database (Table 6) provided further support for the hematological toxicity of TMZ, identifying strong signals for thrombocytopenia (*N* = 9, *Ω* = 0.409), neutropenia (*N* = 5, *Ω* = 0.637), and lymphopenia (*N* = 3, *Ω* = 0.977), with the latter two rated as high priority.

**Table 5:** Analysis of immune-related adverse event signals of temozolomide in the FAERS database.

Adverse event	*N*	*Ω*	95% CI	PRR	ROR	*Ω* Sig	Consistency	Priority
Thrombocytopenia	402	0.088	(1.22–1.56)	Yes	Yes	Yes	Strong	Medium
Neutropenia	177	0.127	(1.02–1.52)	Yes	Yes	Yes	Strong	Medium
Pancytopenia	149	0.189	(1.76–2.5)	Yes	Yes	Yes	Strong	High
Lymphopenia	77	0.196	(0.94–1.71)	Yes	Yes	Yes	Strong	Medium
Sepsis	68	0.193	(0.7–1.46)	Yes	Yes	Yes	Strong	Medium
Febrile neutropenia	66	0.240	(1.22–2.16)	Yes	Yes	Yes	Strong	High
Aspartate aminotransferase increased	32	0.239	(0.01–0.94)	No	Yes	Yes	Moderate	Low
Aplastic anaemia	24	0.841	(1.79–5.09)	Yes	Yes	Yes	Strong	High
Toxic epidermal necrolysis	16	0.589	(1.04–3.35)	Yes	Yes	Yes	Strong	High
Hepatitis	15	0.373	(0.05–1.51)	Yes	Yes	Yes	Strong	Medium
Pleural effusion	13	0.600	(0.82–3.17)	Yes	Yes	Yes	Strong	High
Drug reaction with eosinophilia and systemic symptoms	10	0.873	(0.88–4.3)	Yes	Yes	Yes	Strong	High
Progressive multifocal leukoencephalopathy	10	0.873	(0.88–4.3)	Yes	Yes	Yes	Strong	High
Herpes simplex encephalitis	9	1.451	(0.75–6.43)	Yes		Yes	Moderate	Low
Lichenoid keratosis	9	0.879	(0.77–4.21)	Yes	Yes	Yes	Strong	High
Eosinophilia	8	0.635	(0.29–2.78)	Yes	Yes	Yes	Strong	High
Erythema multiforme	7	0.730	(0.31–3.18)	Yes	Yes	Yes	Strong	High
Diabetes insipidus	7	0.895	(0.5–4.01)	Yes	Yes	Yes	Strong	High

**Table 6:** Analysis of immune-related adverse event signals of temozolomide in the CVARD database.

Adverse event	*N*	*Ω*	95% CI	PRR	ROR	*Ω* Sig	Consistency	Priority
Thrombocytopenia	9	0.409	(0.7–2.3)	Yes	Yes	Yes	Strong	Medium
Neutropenia	5	0.637	(1–3.5)	Yes	Yes	Yes	Strong	High
Herpes simplex encephalitis	4	1.491	(1.33–7.17)	Yes		Yes	Moderate	Low
Lymphopenia	3	0.977	(0.98–4.81)	Yes	Yes	Yes	Strong	High

Bevacizumab and temozolomide combination therapy: In the FAERS database (Table 7), the high-priority signals associated with the combination treatment regimen were primarily concentrated in severe immunosuppression and opportunistic infections. Although diarrhoea (*N* = 33, *Ω* = 0.200) and lymphocyte count decreased (*N* = 23, *Ω* = 0.237) had relatively high reporting frequencies, their signal strength and consistency were rated as medium or low priority. The events warranting the highest vigilance were a series of serious events with low report counts but exceptionally high signal strength (all *Ω* values >0.6) and “strong” consistency: Haemophagocytic lymphohistiocytosis (*N* = 7, *Ω* = 0.648): This is a life-threatening systemic inflammatory response syndrome with a very strong signal. Strongyloidiasis (*N* = 6, *Ω* = 0.744): The strong signal for this opportunistic infection suggests that combination therapy may induce immunosuppression, leading to parasitic dissemination. Epstein–Barr virus infection (*N* = 6, *Ω* = 0.580): Associated with viral reactivation. Pneumonia cytomegaloviral (*N* = 4, *Ω* = 0.943): The *Ω* value for this event is close to 1, indicating a very high signal strength. Nephrotic syndrome (*N* = 3, *Ω* = 0.756): Suggests a potential nephrotoxicity signal. The analysis of the CVARD database (Table 8) partially corroborated the FAERS findings, providing further support for specific signals of the combination therapy. Among these, nephrotic syndrome (*N* = 4, *Ω* = 0.551) re-emerged and was rated as a strong signal of medium priority. Concurrently, Epstein–Barr virus infection (*N* = 6, *Ω* = 1.468) and pneumonia cytomegaloviral (*N* = 6, *Ω* = 1.468), although not frequently reported, exhibited extremely high *Ω* values (>1.4) in this database, despite their consistency being rated as “medium.”

**Table 7:** Analysis of immune-related adverse event signals for the combination therapy of bevacizumab and temozolomide in the FAERS database.

Adverse event	*N*	*Ω*	95% CI	PRR	ROR	*Ω* Sig	Consistency	Priority
Diarrhoea	33	0.200	(0.29–1.07)	No	Yes	Yes	Moderate	Low
Lymphocyte count decreased	23	0.237	(0.15–1.08)	No	Yes	Yes	Moderate	Low
Haemophagocytic lymphohistiocytosis	7	0.648	(1.27–3.81)	Yes	Yes	Yes	Strong	High
Strongyloidiasis	6	0.744	(1.27–4.19)	Yes	Yes	Yes	Strong	High
Epstein–Barr virus infection	6	0.580	(0.81–3.08)	Yes	Yes	Yes	Strong	High
Pneumonia cytomegaloviral	4	0.943	(1.03–4.72)	Yes	Yes	Yes	Strong	High
Nephrotic syndrome	3	0.756	(0.29–3.26)	Yes	Yes	Yes	Strong	High
Enterocolitis	3	0.713	(0.13–2.92)	Yes	No	Yes	Moderate	Low

**Table 8:** Analysis of immune-related adverse event signals for the combination therapy of bevacizumab and temozolomide in the CVARD database.

Adverse event	*N*	*Ω*	95% CI	PRR	ROR	*Ω* Sig	Consistency	Priority
Epstein–Barr virus infection	6	1.468	(2.04–7.79)	Yes		Yes	Moderate	Low
Pneumonia cytomegaloviral	6	1.468	(2.04–7.79)	Yes		Yes	Moderate	Low
Nephrotic syndrome	4	0.551	(0.25–2.41)	Yes	Yes	Yes	Strong	Medium

Comprehensive analysis indicates that BEV + TMZ combination therapy was associated with disproportionate reporting of severe immunosuppression, opportunistic infections, and virus reactivation–related events in spontaneous reporting databases. Compared to the hematological toxicities commonly observed with monotherapies, events associated with the combination therapy, such as haemophagocytic lymphohistiocytosis, strongyloidiasis, and pneumonia cytomegaloviral, although limited in absolute report numbers, are characterized by exceptionally strong signal strengths (high *Ω* values) and partial cross-database validation. This strongly suggests that these represent potential serious safety signals requiring extremely high attention and close monitoring during combination treatment. In clinical practice, heightened vigilance for the aforementioned infectious and immune-related complications is warranted in patients subjected to this combination regimen.

### 3.3 Time-to-onset (TTO) analysis of immune-related adverse events across different treatment groups

In the BEV monotherapy group, significant differences in TTO were observed among various adverse events. Hematological events such as decreased lymphocyte count (*N* = 13, median TTO 34.0 days) and decreased neutrophil count (*N* = 5, median TTO 6.0 days) occurred relatively early, with the latter exhibiting an especially short latency period. In contrast, cholangitis (*N* = 5, median TTO 293.0 days) and optic neuritis (*N* = 1, TTO 483.0 days) demonstrated markedly late onset, suggesting that these are delayed adverse events. Arthralgia (*N* = 12, median TTO 14.0 days) and pruritus (*N* = 4, median TTO 14.0 days) occurred during the early phase.

The time-to-onset (TTO) analysis for the TMZ monotherapy group encompassed a wide range of hematological and non-hematological events. Core hematological toxicities, such as thrombocytopenia (*N* = 177, median TTO 32.0 days), neutropenia (*N* = 57, median TTO 34.0 days), and febrile neutropenia (*N* = 41, median TTO 34.0 days), predominantly occurred within approximately the first month following treatment initiation. In contrast, aplastic anaemia (*N* = 26, median TTO 250.5 days) demonstrated a notably delayed onset, with a median time exceeding 8 months. Severe cutaneous adverse reactions, such as toxic epidermal necrolysis (*N* = 8, median TTO 78.0 days), and neurological events, such as progressive multifocal leukoencephalopathy (*N* = 3, median TTO 153.0 days), also exhibited median-to-late onset patterns. Hepatotoxic events, including increased aspartate aminotransferase (*N* = 26, median TTO 37.5 days), occurred at times similar to those of the hematological events.

The TTO data for the combination therapy group were relatively limited but revealed several meaningful patterns. The onset of lymphocyte count decreased (*N* = 13, median TTO 23.0 days) occurred earlier than that in the BEV monotherapy group (34.0 days). Similarly, diarrhoea (*N* = 6, median TTO 21.5 days) also exhibited an earlier onset trend in the combination therapy. Notably, the severe infection event pneumonia cytomegaloviral (*N* = 3, median TTO 14.0 days) occurred very early, suggesting the need for heightened vigilance for such events during the initial phase of combination therapy. In summary, adverse events associated with BEV monotherapy demonstrated a biphasic distribution, encompassing both early-onset hematological and general events, as well as delayed severe events with extremely long latency periods (e.g., cholangitis, optic neuritis). The myelosuppressive toxicity of TMZ monotherapy was primarily concentrated in the early treatment phase (approximately 1 month), yet it may induce certain severe late sequelae (e.g., aplastic anaemia, progressive multifocal leukoencephalopathy). BEV + TMZ combination therapy was associated with a shorter reported TTO for some events (e.g., lymphopenia and diarrhoea) and with early reporting of severe viral infections (e.g., cytomegaloviral pneumonia) (Table 9).

**Table 9:** Time-to-onset (TTO) analysis of key immune-related adverse events across treatment groups.

Treatment group	Preferred term	Events (*n*)	Median TTO (days)	IQR (days)	Min (days)	Max (days)
BEV monotherapy	Diarrhoea	14	43.0	19.5–71	1	594
BEV monotherapy	Lymphocyte count decreased	13	34.0	13–44	6	77
BEV monotherapy	Arthralgia	12	14.0	12.2–33	1	207
BEV monotherapy	Cholangitis	5	293.0	293–293	27	293
BEV monotherapy	Neutrophil count decreased	5	6.0	3–16	3	19
BEV monotherapy	Pruritus	4	14.0	11.8–31.8	5	85
BEV monotherapy	Optic neuritis	1	483.0	483–483	483	483
BEV + TMZ combination	Lymphocyte count decreased	13	23.0	9–43	3	450
BEV + TMZ combination	Diarrhoea	6	21.5	2–60.5	2	108
BEV + TMZ combination	Pneumonia cytomegaloviral	3	14.0	14–14	14	14
BEV + TMZ combination	Enterocolitis	1	24.0	24–24	24	24
TMZ monotherapy	Thrombocytopenia	177	32.0	23–48	1	637
TMZ monotherapy	Pancytopenia	92	32.5	23.8–46.2	2	667
TMZ monotherapy	Sepsis	65	51.0	27–201	1	571
TMZ monotherapy	Neutropenia	57	34.0	21–58	3	416
TMZ monotherapy	Febrile neutropenia	41	34.0	31–42	19	404
TMZ monotherapy	Aplastic anaemia	26	250.5	74.2–440.8	20	571
TMZ monotherapy	Aspartate aminotransferase increased	26	37.5	17.5–74	1	252
TMZ monotherapy	Lymphopenia	26	41.5	20.2–67.5	5	748
TMZ monotherapy	Hepatitis	10	43.0	33.2–58	7	637
TMZ monotherapy	Lichenoid keratosis	9	34.0	34–34	34	62
TMZ monotherapy	Toxic epidermal necrolysis	8	78.0	42.5–92.2	1	131
TMZ monotherapy	Eosinophilia	7	40.0	7–49.5	3	55
TMZ monotherapy	Pleural effusion	6	34.0	22.5–56.8	6	276
TMZ monotherapy	Drug reaction with eosinophilia and systemic symptoms	5	24.0	9–54	2	66
TMZ monotherapy	Progressive multifocal leukoencephalopathy	3	153.0	94.5–184	36	215
TMZ monotherapy	Diabetes insipidus	2	28.5	21.8–35.2	15	42
TMZ monotherapy	Erythema multiforme	1	79.0	79–79	79	79
TMZ monotherapy	Herpes simplex encephalitis	1	39.0	39–39	39	39

IQR, interquartile range; TTO, time-to-onset (days).

These TTO results are based on the subset of reports with complete date information (*N* shown for each event in Table 9) and should be interpreted as descriptive, report-based onset-time distributions rather than population-level median times or incidence-based estimates.

### 3.4 Exploratory Weibull shape analysis of reported TTO distributions

To further characterize the timing of reported immune-related adverse events (irAEs) beyond median TTO, we fitted an exploratory Weibull distribution to the TTO data for events with adequate report counts (pre-specified *N* ≥ 10 within each regimen). The Weibull shape parameter (*β*) was used solely as a descriptive indicator of whether reported onsets tended to cluster later (*β* > 1.2), approximately uniformly over time (*β* = 0.8–1.2), or earlier (*β* < 0.8). Because spontaneous reporting systems do not provide follow-up time for exposed patients without the event, these fits should not be interpreted as population-level hazard functions, and estimates may be unstable under sparse reporting (Table 10).

**Table 10:** Weibull distribution analysis of time dependency for immune-related adverse events across treatment groups.

Treatment group	Preferred term	*N*	Shape paramete*r* (*β*)	Scale paramete*r* (*η*)	Risk pattern
BEV monotherapy	Diarrhoea	14	0.725	75.872	Decreasing
BEV monotherapy	Lymphocyte count decreased	13	1.501	35.871	Increasing
BEV monotherapy	Arthralgia	12	0.793	30.364	Decreasing
BEV monotherapy	Cholangitis	5	2.114	263.851	Increasing
BEV monotherapy	Neutrophil count decreased	5	1.415	10.385	Increasing
BEV monotherapy	Pruritus	4	0.967	28.999	Constant
BEV + TMZ combination	Lymphocyte count decreased	13	0.717	44.736	Decreasing
BEV + TMZ combination	Diarrhoea	6	0.653	28.000	Decreasing
TMZ monotherapy	Thrombocytopenia	177	1.004	53.359	Constant
TMZ monotherapy	Pancytopenia	92	1.033	50.025	Constant
TMZ monotherapy	Sepsis	65	0.814	126.837	Constant
TMZ monotherapy	Neutropenia	57	0.956	55.348	Constant
TMZ monotherapy	Febrile neutropenia	41	1.179	54.136	Constant
TMZ monotherapy	Aspartate aminotransferase increased	26	1.036	54.031	Constant
TMZ monotherapy	Lymphopenia	26	0.816	70.830	Constant
TMZ monotherapy	Aplastic anaemia	26	1.175	277.231	Constant
TMZ monotherapy	Hepatitis	10	0.706	117.602	Decreasing
TMZ monotherapy	Lichenoid keratosis	9	3.794	40.677	Increasing
TMZ monotherapy	Toxic epidermal necrolysis	8	1.138	71.704	Constant
TMZ monotherapy	Eosinophilia	7	1.166	31.649	Constant
TMZ monotherapy	Pleural effusion	6	0.861	66.016	Constant
TMZ monotherapy	Drug reaction with eosinophilia and systemic symptoms	5	1.034	31.390	Constant
TMZ monotherapy	Progressive multifocal leukoencephalopathy	3	1.821	151.018	Increasing

Risk pattern classification: increasing (shape parameter *β* > 1.2), constant (*β* = 0.8–1.2), decreasing (*β* < 0.8).

In the BEV monotherapy group, some events showed later-clustering of reported onset times (e.g., lymphocyte count decreased, neutrophil count decreased, and cholangitis with *β* > 1.2), whereas others showed earlier-clustering (e.g., diarrhoea and arthralgia with *β* < 0.8). In the TMZ monotherapy group, several hematologic toxicities had *β* values near 1, suggesting a relatively stable distribution of reported onsets, while a small number of events exhibited later-clustering (e.g., progressive multifocal leukoencephalopathy). In the combination therapy group, Weibull modeling was only interpreted where the report-count threshold was met; therefore, no strong inference is made about between-regimen differences in *β*, and any apparent differences should be regarded as hypothesis-generating and potentially influenced by sparse counts and reporting heterogeneity.

Overall, these exploratory Weibull fits are intended to complement the descriptive TTO summaries (Table 9) and to provide a qualitative description of reported onset-time clustering rather than definitive evidence of “changed” temporal risk patterns.

### 3.5 Signal analysis of drug–drug interaction patterns in combination therapy

To explore potential interaction signals in spontaneous reporting data, we evaluated PRR (combination vs. each monotherapy) and the interaction OR as measures of more-than-additive reporting disproportionality. Several events yielded very large interaction OR estimates; however, in spontaneous reporting systems such extreme values may arise when one or more cells in the underlying 2 × 2 table are small, leading to unstable denominators. Accordingly, we interpret these findings as hypothesis-generating interaction signals and do not interpret the magnitude of extreme interaction ORs as effect sizes or evidence of pharmacodynamic synergy.

In the FAERS database (Table 11), compared to BEV monotherapy, the combination therapy demonstrated PRR values greater than 2 for thrombocytopenia (PRR = 2.99), neutropenia (PRR = 5.23), pancytopenia (PRR = 12.86), lymphopenia (PRR = 3.39), febrile neutropenia (PRR = 2.14), and enterocolitis (PRR = 3.67), indicating higher reporting of these events in the combination group. Several events also exhibited very large interaction OR estimates, suggesting possible more-than-additive reporting patterns; however, such extreme values can be inflated by sparse cell counts and unstable denominators in spontaneous reporting systems, and therefore should be interpreted as hypothesis-generating signals rather than evidence of pharmacodynamic synergy. A broadly similar pattern was observed in the CVARD database (Table 12), where the combination therapy also showed elevated PRRs for thrombocytopenia (PRR = 2.21) and neutropenia (PRR = 8.86). Notably, some interaction OR estimates were extremely large, which should be interpreted cautiously as potential sparse-data artifacts despite qualitative consistency of elevated PRR patterns.

**Table 11:** Potential drug–drug interaction signals (more-than-additive reporting disproportionality patterns) for BEV + TMZ versus BEV in the FAERS database.

Adverse event	PRR (combination vs. BEV)	PRR (combination vs. TMZ)	Interaction OR
Thrombocytopenia	2.99	0.49	200.94
Neutropenia	5.23	0.63	810.95
Pancytopenia	12.86	0.33	2,370
Lymphopenia	3.39	0.56	1,214
Febrile neutropenia	2.14	0.25	894.6
Enterocolitis	3.67	7.12	101,726

PRR = proportional reporting ratio; OR = odds ratio; BEV = bevacizumab; TMZ = temozolomide. Interaction ORs in spontaneous reporting data can be highly unstable under sparse cell counts and should be interpreted as hypothesis-generating reporting interaction signals, not as incidence estimates, effect sizes, or confirmed pharmacodynamic synergy.

**Table 12:** Potential drug–drug interaction signals (more-than-additive reporting disproportionality patterns) for BEV + TMZ versus BEV in the CVARD database.

Adverse event	PRR (combination vs. BEV)	PRR (combination vs. TMZ)	Interaction OR
Thrombocytopenia	2.21	0.43	455.69
Neutropenia	8.86	0.52	3,294

PRR = proportional reporting ratio; OR = odds ratio; BEV = bevacizumab; TMZ = temozolomide. Interaction ORs in spontaneous reporting data can be highly unstable under sparse cell counts and should be interpreted as hypothesis-generating reporting interaction signals, not as incidence estimates, effect sizes, or confirmed pharmacodynamic synergy.

In the FAERS database (Table 13), compared with TMZ monotherapy, the combination therapy showed elevated reporting for several non-hematologic events (e.g., diarrhoea PRR = 3.92; lymphocyte count decreased PRR = 4.55; arthralgia PRR = 2.64), with corresponding large interaction OR estimates. Enterocolitis showed increased PRRs in both comparisons (combination vs. BEV: PRR = 3.67; combination vs. TMZ: PRR = 7.12) along with an extremely large interaction OR estimate; this pattern prioritizes enterocolitis as a potential interaction signal, while recognizing that the magnitude of the interaction OR may be unstable in sparse data settings.

**Table 13:** Potential drug–drug interaction signals (more-than-additive reporting disproportionality patterns) for BEV + TMZ versus TMZ in the FAERS database.

Adverse event	PRR (combination vs. BEV)	PRR (combination vs. TMZ)	Interaction OR
Diarrhoea	1.35	3.92	1860
Lymphocyte count decreased	0.97	4.55	2,237
Arthralgia	0.66	2.64	2032
Enterocolitis	3.67	7.12	101,726
Optic neuritis	0.41	2.37	11,298

PRR = proportional reporting ratio; OR = odds ratio; BEV = bevacizumab; TMZ = temozolomide. Interaction ORs in spontaneous reporting data can be highly unstable under sparse cell counts and should be interpreted as hypothesis-generating reporting interaction signals, not as incidence estimates, effect sizes, or confirmed pharmacodynamic synergy.

Overall, the interaction analysis suggests that BEV + TMZ co-reporting may be associated with more-than-additive reporting disproportionality patterns for selected hematologic and non-hematologic toxicities. These findings are intended to prioritize potential safety signals for clinical vigilance and further validation, and do not quantify incidence or establish causality.

### 3.6 Multivariate logistic regression analysis of risk factors for immune-related adverse events (IRAEs)

A multivariable logistic regression model was used to assess factors associated with IRAE reporting after adjustment for sex, age group, body-weight group, and reporter type (Table 14). The model showed acceptable fit (McFadden *R*^2^ = 0.1069; AIC = 122.64; BIC = 152.93). BEV monotherapy was associated with higher odds of IRAE reporting compared with TMZ monotherapy (OR = 3.6037, 95% CI: 1.0378–12.652, *p* < 0.05). In contrast, the overall IRAE reporting odds for BEV + TMZ were not statistically different from TMZ monotherapy (OR = 2.9677, 95% CI: 0.5018–15.2056, *p* = 0.1988) or BEV monotherapy (OR = 0.8235, 95% CI: 0.1454–4.6642, *p* = 0.8263). Sex, age group, and body-weight group were not significantly associated with IRAE reporting; the >100 kg category was not estimable due to sparse data and (quasi-)complete separation. Reporter type showed heterogeneity, with OT vs. MD demonstrating increased reporting odds (OR = 1.7803, 95% CI: 0.4897–6.2832), whereas other reporter categories were not statistically significant.

**Table 14:** Multivariable logistic regression analysis of risk factors for immune-related adverse events.

Variables	OR	95% CI	*p* value	Signif.
Intercept	0.1056	0.0288–0.3226	<0.001	***
Sex (female vs. male)	1.6477	0.5975–4.7627	0.3403	ns
Age Group (≥65y vs. <65y)	1.0752	0.3485–3.2129	0.8967	ns
Weight group (<50 kg vs. 50–100 kg)	1.4654	0.1473–11.2772	0.7212	ns
Weight group (>100 kg vs. 50–100 kg)	0	Not estimable	0.9901	ns
Drug (BEV mono vs. TMZ mono)	3.6037	1.0378–12.652	<0.05	*
Drug (BEV + TMZ combo vs. TMZ mono)	2.9677	0.5018–15.2056	0.1988	ns
Reporter type (OT vs. MD)	1.7803	0.4897–6.2832	0.3684	ns
Reporter type (HP vs. MD)	1.0685	0.1402–5.4981	0.941	ns
Reporter type (CN vs. MD)	1.461	0.2729–6.9018	0.6386	ns
Reporter type (PH vs. MD)	0.6058	0.03–4.2041	0.6614	ns
Drug (BEV + TMZ combo vs. BEV mono)	0.8235	0.1454–4.6642	0.8263	ns
Model fit indices				
McFadden *R*^2^	0.1069			
AIC	122.64			
BIC	152.93			

IRAE = immune-related adverse event; OR = odds ratio; CI = confidence interval. ***p < 0.001, *p < 0.05, ns = not significant. Reference groups: sex = male; age group = <65 years; weight group = 50–100 kg; drug regimen = TMZ monotherapy; reporter type = MD. NE indicates the OR/CI was not estimable due to sparse data and (quasi-)complete separation in the logistic model.

## 4 Discussion

This study conducted a systematic pharmacovigilance investigation of immune-related adverse events (IRAEs) associated with bevacizumab (BEV), temozolomide (TMZ) monotherapy, and their combination therapy in patients with glioblastoma, based on two major global pharmacovigilance databases (FAERS and CVARD). Our analysis revealed several key and novel findings. First, the BEV and TMZ combination therapy exhibited a distinct safety profile characterized primarily by profound immunosuppression and opportunistic infections, which is qualitatively different from the features observed with either monotherapy. Second, the drug interaction analysis identified hypothesis-generating more-than-additive reporting disproportionality patterns for selected toxicities in spontaneous reporting data, which should be interpreted as potential interaction signals rather than confirmed pharmacodynamic synergy. Finally, this study provides a comprehensive delineation of time-to-onset and onset-hazard patterns for key IRAEs and suggests that BEV + TMZ combination therapy is associated with higher IRAE reporting odds in spontaneous reporting databases.

### 4.1 Unique safety profile of combination therapy: from hematologic toxicity to immunosuppression and opportunistic infections

The core finding of this study is the identification of a strong association between the BEV + TMZ combination therapy and a series of rare yet extremely severe immune-related adverse events. Unlike TMZ monotherapy, which primarily induces classic myelosuppression such as thrombocytopenia and neutropenia, the most significant signals in the combination therapy group included hemophagocytic lymphohistiocytosis (HLH), strongyloidiasis, and Epstein–Barr virus (EBV) or cytomegalovirus (CMV) reactivation. HLH is a life-threatening systemic inflammatory storm, the occurrence of which is typically associated with severe immune dysregulation (16). The emergence of opportunistic infections, such as strongyloidiasis and CMV pneumonia, suggests that the combination treatment may induce a profound, comprehensive state of immunodeficiency that extends beyond the known lymphopenia effect of TMZ (17). The underlying mechanism is likely multifaceted. Specifically, by inhibiting VEGF, BEV not only targets angiogenesis but also disrupts the role of VEGF in maintaining immune homeostasis, including affecting dendritic cell maturation, T-cell function, and lymphocyte homing (18). When this VEGF-mediated perturbation of the immune microenvironment is combined with the lymphocytotoxic effects of TMZ, the body’s ability to control latent pathogens and maintain immune tolerance balance may be significantly compromised, thereby creating conditions conducive to HLH and opportunistic infections. Clinicians must be aware that the risks associated with combination therapy have expanded from traditional myelosuppression to broader immune dysfunction. In patients presenting with unexplained fever, pancytopenia, or pneumonia, these rare but fatal complications should be actively investigated.

### 4.2 More-than-additive disproportionality signals: potential interaction signals in spontaneous reporting data

Our drug interaction analysis provides quantitative support for potential interaction signals manifesting as more-than-additive reporting disproportionality, as combination therapy showed elevated PRRs and large interaction OR estimates for multiple toxicities under our pre-specified prioritization criteria; however, extremely large interaction ORs may reflect sparse data and unstable denominators, and should be interpreted cautiously as hypothesis-generating signals rather than definitive pharmacodynamic synergy. This suggests that the combination of BEV and TMZ may exhibit a “more-than-additive” pattern in spontaneous reporting data (i.e., a “1 + 1 > 2” reporting effect). The biological basis for this pattern may lie in the fact that TMZ-induced myelosuppression and damage to the intestinal mucosal barrier create a susceptible environment for BEV’s actions (10). Conversely, BEV could further contribute to TMZ-associated toxicity by inhibiting vascular endothelial repair and exacerbating tissue hypoxia and inflammation (19). For instance, the remarkable more-than-additive reporting pattern observed for enteritis likely stems from the combined action of TMZ’s direct damage to intestinal epithelial cells and BEV’s impairment of ulcer healing (20), ultimately predisposing to severe transmural inflammation and infection. Similarly, for events such as optic neuritis, BEV may increase susceptibility to TMZ or other immune-mediated damage by interfering with the blood supply to the optic nerve (21). Importantly, these findings from spontaneous reporting data should be interpreted as hypothesis-generating signals rather than definitive evidence of pharmacodynamic synergy or causal toxicity. These signals caution that the safety profile of combination regimens may not be fully inferred from the safety profiles of individual drugs in clinical practice. It is prudent to communicate these potential interaction signals and to consider heightened clinical vigilance, while recognizing that prospective studies are needed to confirm causality and quantify incidence.

### 4.3 Temporal patterns of reported events: a time-to-onset perspective for signal-informed vigilance

Our time-to-onset analyses summarize the reported timing of IRAEs among cases with available date information, complementing disproportionality signals by describing when reported events tend to occur (Table 9). Several hematologic events were reported relatively early after treatment initiation, whereas some events showed later reported onsets. In the combination group, lymphocyte count decreased was reported with a shorter median TTO than in BEV monotherapy, and cytomegaloviral pneumonia—although rare and based on very few reports—was reported early (median 14 days), highlighting the need to remain vigilant for opportunistic infections from treatment initiation. Exploratory Weibull fits (when report counts permitted) were used only to describe the shape of reported onset-time distributions and should not be interpreted as population-level hazard functions or definitive evidence of altered temporal risk patterns.

### 4.4 Risk factors and clinical implications

In the adjusted analysis, BEV monotherapy showed higher IRAE reporting odds compared with TMZ monotherapy (OR = 3.6037, 95% CI: 1.0378–12.652, *p* < 0.05). The overall IRAE reporting odds for the BEV + TMZ regimen were not significantly different from either monotherapy after adjustment, underscoring that spontaneous-report–based regression reflects reporting propensity rather than causal risk. Demographic variables (sex, age group, and body-weight group) were not statistically significant in the adjusted model; notably, the >100 kg category was not estimable because of sparse data/separation. We therefore interpret these covariates cautiously and emphasize that residual confounding (including severity, dosing intensity, and treatment discontinuation) and reporting heterogeneity remain possible.

### 4.5 Study strengths and limitations

The strengths of this study include the use of large-scale, international real-world data and a multi-algorithm triangulation strategy to enhance signal robustness, providing a comprehensive assessment of the interaction patterns, time-to-onset characteristics, and factors associated with reported IRAEs under BEV + TMZ combination therapy. Nevertheless, several limitations should be acknowledged. First, as a spontaneous reporting system, FAERS is subject to under-reporting, reporting bias, and incomplete clinical information. Second, key patient-level variables (e.g., tumor stage, prior therapies, concomitant medications, and laboratory results) were unavailable, and residual confounding cannot be excluded. Third, disproportionality measures (e.g., PRR, ROR) reflect reporting associations (signals) rather than incidence or causal risk; causal inference requires prospective validation. Indication/diagnosis fields and drug-role assignments in spontaneous reports may be incomplete or miscoded, which can introduce cohort and exposure misclassification; therefore, we performed term-dictionary specification (Supplementary Table S1) and an any-role exposure sensitivity analysis (Supplementary Table S2) to assess robustness. In addition, confounding by indication and disease severity (channeling bias) may affect reporting patterns because patients receiving combination therapy may systematically differ from monotherapy recipients. Onset-date and treatment start-date fields are frequently missing in spontaneous reports; because follow-up time for exposed patients without the event is not observed, censoring cannot be defined and survival analyses (e.g., Kaplan–Meier/Cox) are not feasible in this setting, and any Weibull-based distribution-shape findings—especially under sparse counts—should be regarded as exploratory. Stimulated reporting driven by heightened awareness, publications, or regulatory communications may further inflate reporting for selected events over time. Moreover, the relatively small number of cases for certain events in CVARD may reduce estimate precision. We also note that high-dimensional screening across many adverse-event terms introduces multiple-testing and signal-multiplicity concerns. Although we employed shrinkage estimation, minimum-count thresholds, and multi-algorithm/cross-database triangulation to mitigate false positives, some findings may still reflect chance variation or correlated term artifacts. Accordingly, all signals should be interpreted as hypothesis-generating and confirmed in clinical and epidemiologic studies.

Finally, our pharmacovigilance framework is complementary to emerging foundation-model approaches for drug response prediction at the single-cell level. Recent studies such as scDrugMap, which benchmarks large foundation models for single-cell drug response prediction, and DrugFormer, which integrates graph-enhanced language modeling to predict drug sensitivity, highlight opportunities to model cellular heterogeneity and treatment response in a mechanistically informed manner (22, 23). Future work could integrate pharmacovigilance-derived safety signals with these response-prediction models to develop hypothesis-generating benefit–risk frameworks that prioritize patient subgroups, event types, and treatment phases for intensified monitoring and prospective validation.

### 4.6 Conclusion

In conclusion, this study systematically elucidated that the bevacizumab and temozolomide combination therapy is associated with a unique spectrum of immune-related adverse events. Clinically, the scope of monitoring should be expanded from traditional hematological toxicities to include active screening for HLH, specific opportunistic infections, and viral pneumonia. We recommend the implementation of an individualized surveillance schedule: focusing on the prevention and early detection of initial events such as diarrhoea and cytomegalovirus infection during the early phase of treatment, maintaining continuous monitoring of blood counts throughout the entire treatment course, and remaining vigilant for late-onset events like cholangitis and PML during long-term therapy and after its completion. Our findings aim to optimize the treatment safety profile for neuro-oncology patients, achieving an optimal balance between therapeutic efficacy and risk management.

## Data Availability

Publicly available datasets were analyzed in this study. This data can be found here: FAERS: https://www.fda.gov/drugs/fdas-adverse-event-reporting-system-faers/fda-adverse-event-reporting-system-faers-public-dashboard; CVARD: https://www.canada.ca/en/health-canada/services/drugs-health-products/medeffect-canada/adverse-reaction-database/canada-vigilance-online-database-data-extract.html.
