# Supplementary material for: A multi-database pharmacovigilance study reveals distinctive immunosuppressive and opportunistic infection disproportionality signals with bevacizumab and temozolomide combination therapy in glioblastoma
Source: Front Med (Lausanne). 2026 Mar 11;13:1773599. doi: 10.3389/fmed.2026.1773599 (PMC13012968; doi:10.3389/fmed.2026.1773599)
Supplement: Supplementary file 1 [file Supplementary_file_1.docx]

**Supplementary Table S1. Indication/diagnosis search terms used to identify glioblastoma reports and definition of a stringent “core GBM” subgroup**

| Category | Search terms (case-insensitive; after text normalization) |
| --- | --- |
| Core GBM terms (used to define the “core GBM” subgroup) | glioblastoma; glioblastoma multiforme; GBM |
| Broad GBM-related terms (used for the primary “broad GBM” cohort) | glioblastoma; glioblastoma multiforme; GBM; malignant glioma; high-grade glioma; glioma, malignant; grade IV glioma; WHO grade IV glioma; gliosarcoma; glioma grade IV; anaplastic glioma |
