# Supplementary material for: A multi-database pharmacovigilance study reveals distinctive immunosuppressive and opportunistic infection disproportionality signals with bevacizumab and temozolomide combination therapy in glioblastoma
Source: Front Med (Lausanne). 2026 Mar 11;13:1773599. doi: 10.3389/fmed.2026.1773599 (PMC13012968; doi:10.3389/fmed.2026.1773599)
Supplement: Supplementary file 2 [file Supplementary_file_2.docx]

**Supplementary Table S2. Sensitivity analysis of key signals using an expanded exposure definition (any-role: PS/SS/C/I) compared with the primary PS-only definition in glioblastoma reports.**

| Database | Key event | Primary exposure (PS-only) N | Primary: signal | Alternative exposure (PS/SS/C/I) N | Alternative: signal | Interpretation |
| --- | --- | --- | --- | --- | --- | --- |
| FAERS | Haemophagocytic lymphohistiocytosis | 7 | Yes | 7 | Yes | Consistent |
| FAERS | Strongyloidiasis | 6 | Yes | 8 | Yes | Consistent |
| FAERS | Epstein–Barr virus infection | 6 | Yes | 6 | Yes | Consistent |
| FAERS | Pneumonia cytomegaloviral | 4 | Yes | 5 | Yes | Consistent |
| FAERS | Nephrotic syndrome | 3 | Yes | 4 | Yes | Consistent |
| FAERS | Pancytopenia | 149 | Yes | 288 | Yes | Consistent |
| FAERS | Neutropenia | 177 | Yes | 275 | Yes | Consistent |
| FAERS | Enterocolitis | 3 | Yes | 4 | Yes | Consistent |
| FAERS | Optic neuritis | 6 | Yes | 6 | Yes | Consistent |
| FAERS | Lymphocyte count decreased | 58 | Yes | 62 | Yes | Consistent |
| CVARD | Lymphocyte count decreased | 52 | Yes | 55 | Yes | Consistent |
| CVARD | Epstein–Barr virus infection | 6 | Yes | 6 | Yes | Consistent |
| CVARD | Pneumonia cytomegaloviral | 6 | Yes | 7 | Yes | Consistent |
| CVARD | Nephrotic syndrome | 4 | Yes | 5 | Yes | Consistent |
| CVARD | Neutropenia | 5 | Yes | 6 | Yes | Consistent |

Signal retained was evaluated using the same signal-detection criteria as the primary analysis (e.g., Ω025 > 0 with minimum report count threshold and/or PRR/ROR screening rules, as specified in the Methods).

PS/SS/C/I indicate drug roles in the report as Primary Suspect, Secondary Suspect, Concomitant, and Interacting, respectively.
